# Oral carbon monoxide therapy in murine sickle cell disease: Beneficial effects on vaso-occlusion, inflammation and anemia

**DOI:** 10.1371/journal.pone.0205194

**Published:** 2018-10-11

**Authors:** John D. Belcher, Edward Gomperts, Julia Nguyen, Chunsheng Chen, Fuad Abdulla, Zachary M. Kiser, David Gallo, Howard Levy, Leo E. Otterbein, Gregory M. Vercellotti

**Affiliations:** 1 University of Minnesota, Department of Medicine, Vascular Research Center, Division of Hematology, Oncology and Transplantation, Minneapolis, MN, United States of America; 2 Hillhurst Biopharmaceuticals, Inc., Montrose, CA, United States of America; 3 Harvard Medical School, Beth Israel Deaconess Medical Center, Boston, MA, United States of America; Massachusetts Institute of Technology, UNITED STATES

## Abstract

Carbon monoxide (CO) at low, non-toxic concentrations has been previously demonstrated to exert anti-inflammatory protection in murine models of sickle cell disease (SCD). However CO delivery by inhalation, CO-hemoglobin infusion or CO-releasing molecules presents problems for daily CO administration. Oral administration of a CO-saturated liquid avoids many of these issues and potentially provides a platform for self-administration to SCD patients. To test if orally-delivered CO could modulate SCD vaso-occlusion and inflammation, a liquid CO formulation (HBI-002) was administered by gavage (10 ml/kg) once-daily to NY1DD and Townes-SS transgenic mouse models of SCD. Baseline CO-hemoglobin (CO-Hb) levels were 1.6% and 1.8% in NY1DD and Townes-SS sickle mice and 0.6% in Townes-AS control mice. CO-Hb levels reached 5.4%, 4.7% and 3.0% within 5 minutes in NY1DD, SS and AS mice respectively after gavage with HBI-002. After ten treatments, each once-daily, hemoglobin levels rose from 5.3g/dL in vehicle-treated Townes-SS mice to 6.3g/dL in HBI-002-treated. Similarly, red blood cell (RBC) counts rose from 2.36 x 10^6^/μL in vehicle-treated SS mice to 2.89 x 10^6^/μL in HBI-002-treated mice. In concordance with these findings, hematocrits rose from 26.3% in vehicle-treated mice to 30.0% in HBI-002-treated mice. Reticulocyte counts were not significantly different between vehicle and HBI-002-treated SS mice implying less hemolysis and not an increase in RBC production. White blood cell counts decreased from 29.1 x 10^3^/μL in vehicle-treated versus 20.3 x 10^3^/μL in HBI-002-treated SS mice. Townes-SS mice treated with HBI-002 had markedly increased Nrf2 and HO-1 expression and decreased NF-κB activation compared to vehicle-treated mice. These anti-inflammatory effects were examined for the ability of HBI-002 (administered orally once-daily for up to 5 days) to inhibit vaso-occlusion induced by hypoxia-reoxygenation. In NY1DD and Townes-SS sickle mice, HBI-002 decreased microvascular stasis in a duration-dependent manner. Collectively, these findings support HBI-002 as a useful anti-inflammatory agent to treat SCD and warrants further development as a therapeutic.

## Introduction

Carbon monoxide (CO) at low, non-toxic concentrations exerts key physiological functions in various models of tissue inflammation and injury, providing potent cytoprotection in models of inflammation including SCD [1–3], organ transplantation [4], and acute lung injury [5], among others [6–8]. The protection observed, both prophylactically and therapeutically, is associated with an inhibition in the inflammatory response and restoration of tissue function, including abrogating ischemia reperfusion injury [9, 10]. CO may also inhibit polymerization of hemoglobin (Hb) S and increase RBC life span [11, 12]. However, delivery systems that include inhaled CO, metallic CO-releasing molecules (CORMs) and CO conjugated to a PEGylated Hb, may not be suitable for the chronic administration of CO in humans that will be necessary to prevent vaso-occlusive crises. Inhaled CO is challenging to precisely dose given the variability in patient ventilation and has environmental safety concerns for patients and bystanders, as it requires the presence of large amounts of compressed CO gas in cylinders. Metal-containing CORMs present potential long-term health concerns [13]. PEGylated CO-Hb is also not appropriate for chronic home use as it would require daily intravenous infusions and could present toxicology challenges [14, 15]. Oral administration of CO avoids many of the challenges associated with inhaled CO, CORMs and PEGylated CO-Hb and additionally provides a platform for outpatient administration and compliance. An oral drug product that would deliver a predictable low, non-toxic dose of CO, might be a better alternative for treating SCD patients. To test the efficacy of oral administration of CO and its anti-inflammatory, anti-vaso-occlusive properties, a liquid CO formulation (HBI-002) was administered by gavage to NY1DD and Townes-SS transgenic mouse models of SCD.

## Materials and methods

### Reagents

HBI-002 is a novel oral drug product consisting of a formulation containing CO as well as FDA-defined Generally Recognized as Safe (GRAS) components and manufactured using a controlled, reproducible process to achieve the targeted CO concentration. Vehicle was the same formulation without CO. Aliquots of HBI-002 and vehicle were supplied by Hillhurst Biopharmaceuticals. HBI-002 and vehicle were administered to mice by gavage.

### Mice

All animal experiments were approved by the University of Minnesota’s Institutional Animal Care and Use Committees. These studies utilized approximately equal numbers of male and female anemic Townes-SS sickle mice and non-anemic Townes-AS control mice on a 129/B6 mixed genetic background [16] and NY1DD sickle mice on a B6 genetic background [17]. Mice were aged 8–12 weeks. The hemoglobin levels in the NY1DD mice are the same as in normal control mice [17] and the hemoglobin levels in the Townes-SS mice are on average 66% of Townes-AS control mice [16].

The Townes-SS mice were created by knocking in human α and ^A^ɣβ^S^ globins into the deletion sites for murine α- and β-globins. The hyperhemolytic Townes-SS mice have anemia and an SS-RBC half-life of 2.5 days (d) [18]. Heterozygous Townes-AS control mice express normal human α globin and one copy each of human ^A^ɣβ^A^ and ^A^ɣβ^S^ globins. The non-anemic AS mice have an 11.5 d RBC half-life [18]. NY1DD sickle mice have linked human α and β^S^ transgenes on a murine β-major thal (del/del) background. NY1DD sickle mice have an RBC half-life of 7 d [18]. Mice were housed in standard size rodent cages with bedding on a 12 hour (h) light/dark cycle at 21°C. All animals were monitored daily including weekends and holidays for health problems, food and water levels and cage conditions. Littermates were randomly assigned to different treatment groups. All animals were included in each endpoint analysis and there were no unexpected adverse events that required modification of the protocol. At the end of the experiments, mice were sacrificed by placing them in a CO_2_ atmosphere.

### Administration of HBI-002

HBI-002 or vehicle was administered once-daily for 1 to 10 d by gavage at a dose of 10 ml/kg. This is equivalent to 0.25 ml in a 25 g mouse.

### Measurement of CO-Hb

Townes-SS and AS mice and NY1DD mice were anesthetized with 10% urethane and 2% α-cholalose (5 ml/kg, ip). EDTA blood samples were collected from the inferior vena cava at the indicated times. Sealed syringes were delivered to the University of Minnesota Hospital clinical lab and CO-Hb was measured within 30 minutes (min) of blood collection using a clinical co-oximeter (Radiometer).

### Measurement of vaso-occlusion (microvascular stasis)

Townes-SS and NY1DD sickle mice were implanted with dorsal skin-fold chambers (DSFCs) as previously described [19]. The same day, mice with DSFCs were anesthetized with ketamine/xylaxine, placed on an intravital microscopy stage, and 20–24 flowing subcutaneous venules were selected and mapped. After baseline selection of venules, mice were challenged with hypoxia-reoxygenation (H/R, 7% O_2_/93% N_2_ for 1h followed by room air). The same vessels selected at baseline were re-examined for stasis (no flow) after 1 h of reoxygenation and percent stasis was calculated.

### Immunoblots

Livers were collected from sickle mice with DSFCs after 4 h of reoxygenation. The livers were flash-frozen in liquid N_2_ and stored at -85°C until use. Microsomes and nuclear extracts were isolated from the livers of mice as previously described [20]. Immunoblots of cellular subfractions were immunostained with primary antibodies to Nrf2 (Proteintech #16396-1-AP), HO-1 (Enzo #ADI-OSA-111), NF-ĸB phospho-p65 (Ser536, Cell Signaling #3031), total p65 (Cell Signaling #3034), VCAM-1 (Abcam #ab174279) and GAPDH (Sigma Aldrich #G9545). Primary antibodies were labeled with the appropriate secondary antibodies conjugated to alkaline phosphatase and visualized with ECF substrate (GE Healthcare) and a Typhoon FLA 9500 scanner (GE Healthcare). Immunoreactive bands on images were quantitated using ImageJ software (NIH). Mean relative expression of protein bands from animals treated with vehicle or HBI-002 groups were calculated.

### Hematology

EDTA blood was collected from the inferior vena cava of Townes-SS mice after 10 days of once-daily gavage with HBI-002 or vehicle. Hb was measured in whole blood in the University of Minnesota Hospital clinical lab. Hematocrit was measured using a micro-capillary reader (IEC) after centrifugation in a micro-capillary centrifuge (IEC). Reticulocytes were counted using blood smears stained with methylene blue. Reticulocytes and total RBC were counted in 4 separate microscopic fields per mouse. Reticulocytes are expressed as a percentage of total RBC. The total white blood cell and RBC counts were counted manually using a hemocytometer.

### Statistics

Analyses were performed with SigmaStat 3.5 for Windows (Systat Software, San Jose, CA). Comparisons of multiple treatment groups were made using one-way analysis of variance (ANOVA) (Holm-Sidak method) or the Student’s t-test as indicated. A Pearson Product Moment Correlation analysis was performed on the number of treatment days versus stasis.

## Results and discussion

**Fig 1:**
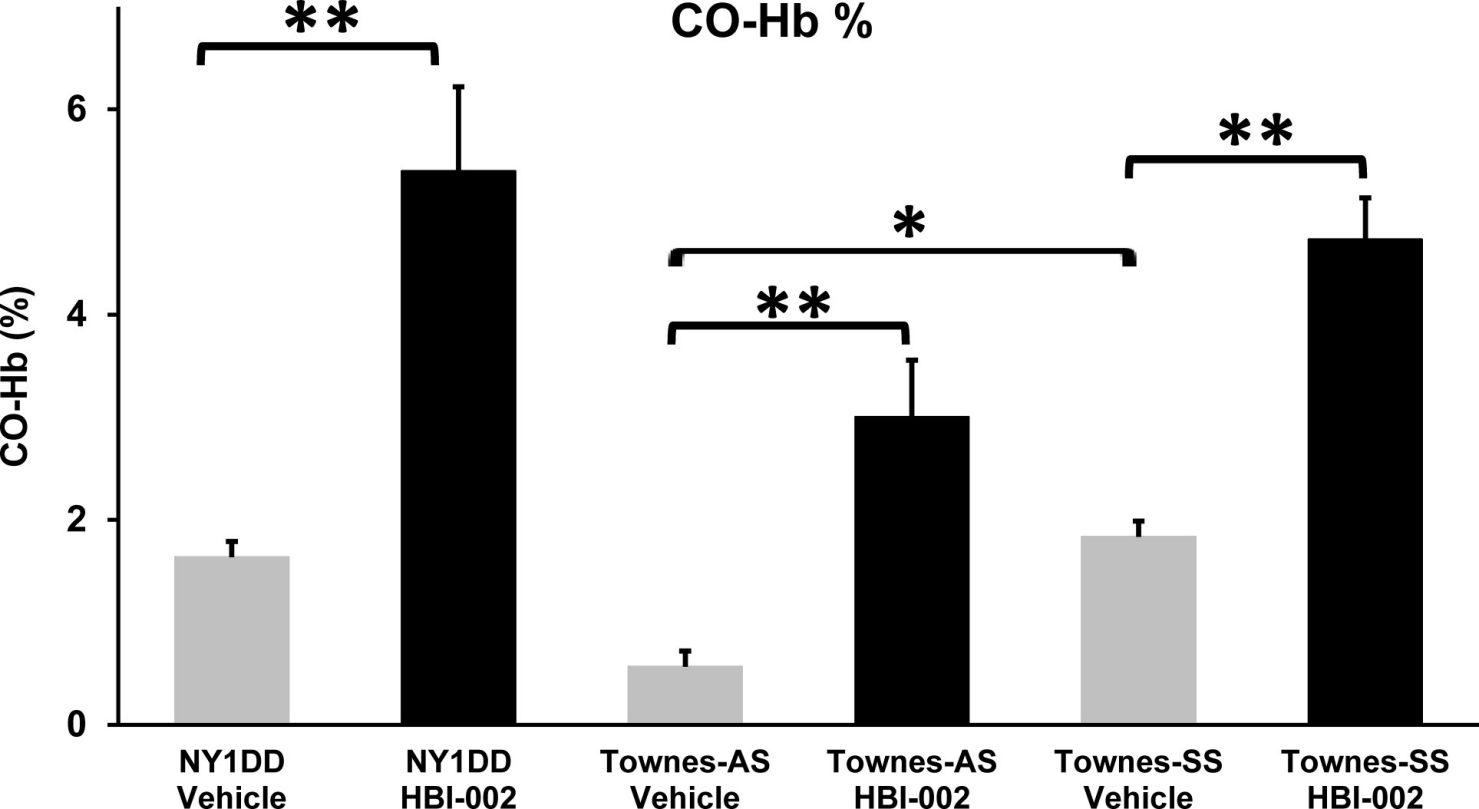
Circulating CO-Hb levels in mice after gavage with HBI-002. NY1DD and Townes-AS and SS mice (n = 3/group) were gavaged once with HBI-002 or vehicle (10 ml/kg) and venous blood was collected 5 min after gavage from the inferior vena cava for measurement of CO-Hb. Bar values represent means + SD. **P<0.01 for vehicle versus HBI-002 and *p<0.05 for Townes-AS versus SS. Differences between groups were analyzed using One Way ANOVA (Holm-Sidak method).

In preliminary studies to examine HBI-002 bioavailability, after a single gavage (10 ml/kg), CO-Hb was cleared from circulation in approximately 3 h in outbred CD-1 mice and peaked between 0–10 min after gavage in Townes-AS mice. Subsequent blood samples were collected 5 min after gavage. In NY1DD sickle mice, CO-Hb levels were 1.6 ± 0.2% (mean ± SD) after treatment with vehicle and increased significantly to 5.4 ± 0.8% after treatment with HBI-002 (p<0.01) **(Fig 1)**. In Townes-AS mice, CO-Hb levels were 0.6 ± 0.2% after gavage with vehicle and increased significantly to 3.0 ± 0.6% after gavage with HBI-002 (p<0.01). Similarly, in Townes-SS mice, CO-Hb levels were 1.8 ± 0.2% (mean ± SD) after treatment with vehicle and increased significantly to 4.7 ± 0.4% after treatment with HBI-002 (p<0.01). Vehicle-treated Townes-AS mice had significantly lower CO-Hb than vehicle-treated Townes-SS mice (p<0.05), likely because of a markedly reduced hemolytic rate in AS mice. This is reflected in significantly shorter RBC half-lives and 6-fold higher expired CO levels in SS versus AS mice [18]. These data document that following oral administration of HBI-002, CO is rapidly absorbed from the gut into the vasculature and is bioavailable in the circulation as measurable CO-Hb within minutes.

**Fig 2:**
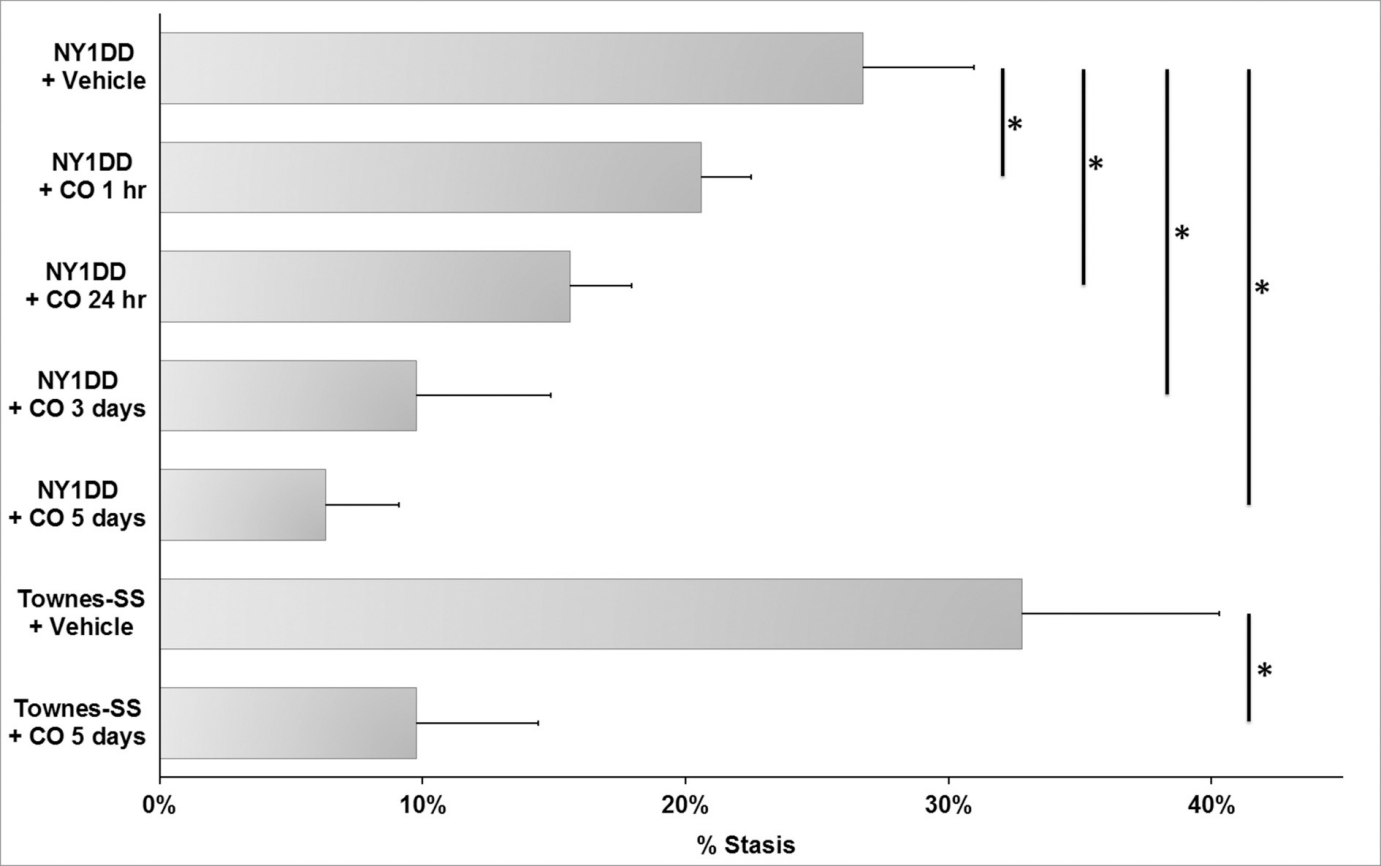
HBI-002 inhibits microvascular stasis. Microvascular stasis was measured in NY1DD and Townes-SS sickle mice (n = 3/bar) with implanted dorsal skin-fold chambers after gavage (10 ml/kg) with CO (HBI-002) or vehicle at 1 h or 24 h before hypoxia (7% O_2_) or once-daily for 3 or 5 days with the last CO treatment 1 h before hypoxia. NY1DD vehicle data from 1, 3 and 5 days were pooled (n = 9 mice). One h prior to hypoxia, flowing venules (20–25 venules/mouse) were selected in the chamber window using intravital microscopy and mapped. After selection of flowing venules, mice were placed in hypoxia for 1 h, and then returned to room air for 1 h (H/R). After H/R the same venules were re-examined for stasis (no flow) and % stasis was calculated. Bar values represent means + SD. *P<0.01. Differences between NY1DD groups were analyzed by One Way ANOVA (Holm-Sidak method). Differences between Townes-SS treatment groups were analyzed by the Student’s t-test.

Microvascular stasis (vaso-occlusion) was measured in sickle mice after exposure to 1 h of hypoxia (7% O_2_) followed by 1 h of reoxygenation in room air (H/R). HBI-002 administered once-daily for 1, 3 or 5 days inhibited H/R-induced microvascular stasis in NY1DD mice versus vehicle control in a duration-dependent manner. Stasis was 27% in vehicle-treated NY1DD mice, 21% when HBI-002 was administered 1 h before H/R and 16% when administered 24 h before H/R. Stasis was 10% after 3 once-daily treatments, and 6% after 5 once-daily treatments **(Fig 2)** with the last CO treatment 1 h before H/R. In Townes-SS mice, H/R-induced stasis was 33% in vehicle-treated mice and 10% after 5 once-daily HBI-002 treatment. A Pearson Product Moment Correlation analysis was performed on the number of treatment days versus stasis. Number of treatment days and stasis had a negative correlation of -0.87 (p<0.001).

The effects of HBI-002 on blood counts were examined in Townes-SS mice treated with HBI-002 (n = 4) or vehicle (n = 3, 10 ml/kg, once-daily X 10 days). Hemoglobin levels increased from 5.3 g/dL in vehicle-treated SS mice to 6.3 g/dL in CO-treated SS mice (p < .05) (**Table 1**). Similarly, RBC counts increased from 2.36 X 10^6^/μL in vehicle-treated SS mice to 2.89 X 10^6^/μL in CO-treated SS mice (p < .05). In concordance with these findings, hematocrits increased from 26.3% in vehicle-treated SS mice to 30.0% in CO-treated SS mice (p < .05). The absolute reticulocyte count was not significantly different in vehicle-treated versus CO-treated SS mice. The percentage of stained F-cells was similar (16%) in both treatment groups. White blood cell counts decreased from 29.1 X 10^3^/μL in vehicle-treated SS mice to 20.3 X 10^3^/μL in CO-treated SS mice (p < .01). The increase in Hb levels, hematocrits and RBC counts without a significant change in the absolute reticulocyte counts (**Table 1**) suggests that CO may have decreased the destruction rate of RBC in SS mice without increasing the production rate of RBC.

**Table 1:** HBI-002 increases hemoglobin, hematocrit and red blood cells and decreases white blood cells in Townes-SS mice.

Treatment	Hemoglobin (g/dL)	Hematocrit (%)	Red Blood Cells (X 10^6^/μL)	Reticulocytes (X 10^6^/μL)	White Blood Cells (X 10^3^/μL)
**Vehicle**	5.30 ± 0.40	26.3 ± 0.8	2.36 ± 0.23	1.32 ± 0.44	29.2 ± 2.4
**HBI-002**	6.34 ± 0.53*	30.0 ± 1.5*	2.89 ± 0.15*	1.42 ± 0.34	20.3 ± 1.7**

Townes-SS mice (n = 4/group) were treated with HBI-002 or vehicle (10ml/kg) 1x/day x 10 days. After 10 days of treatment, venous blood was collected and complete blood counts were measured. Bar values represent means ± SD.

*P<0.05 and

**p<0.01, HBI-002 vs. vehicle.

Differences between vehicle and HBI-002 were analyzed using the Student’s t-test.

Is an increased hematocrit beneficial? Some published reports indicate a higher hemoglobin/hematocrit level is associated with higher rates of pain in SCD. For example in the Cooperative Study of Sickle Cell Disease, concurrent α thalassemia decreases hemolysis, but was associated with a viscosity phenotype of acute vaso-occlusive painful episodes [21]. Another study of the same cohort reported a higher baseline hematocrit was associated with 1 or more painful crises [22]. However, a detailed study of biomarker clusters in this cohort found patients with less intravascular hemolysis had a reduction in pain and acute chest syndrome in addition to a trend to reduced mortality and stroke [23]. High hemoglobin F levels have also been associated with reduced incidence of acute painful episodes [21]. In addition, hyperhemolysis occurs during uncomplicated acute painful episodes in some patients with SCD [24]. Finally, there are multiple publications indicating that a lower hemoglobin/hematocrit is associated with greater severity of SCD [25–28]. Thus, the link between hematocrit and morbidity in SCD is not always clear and may be associated with subphenotypes of the disease.

**Fig 3:**
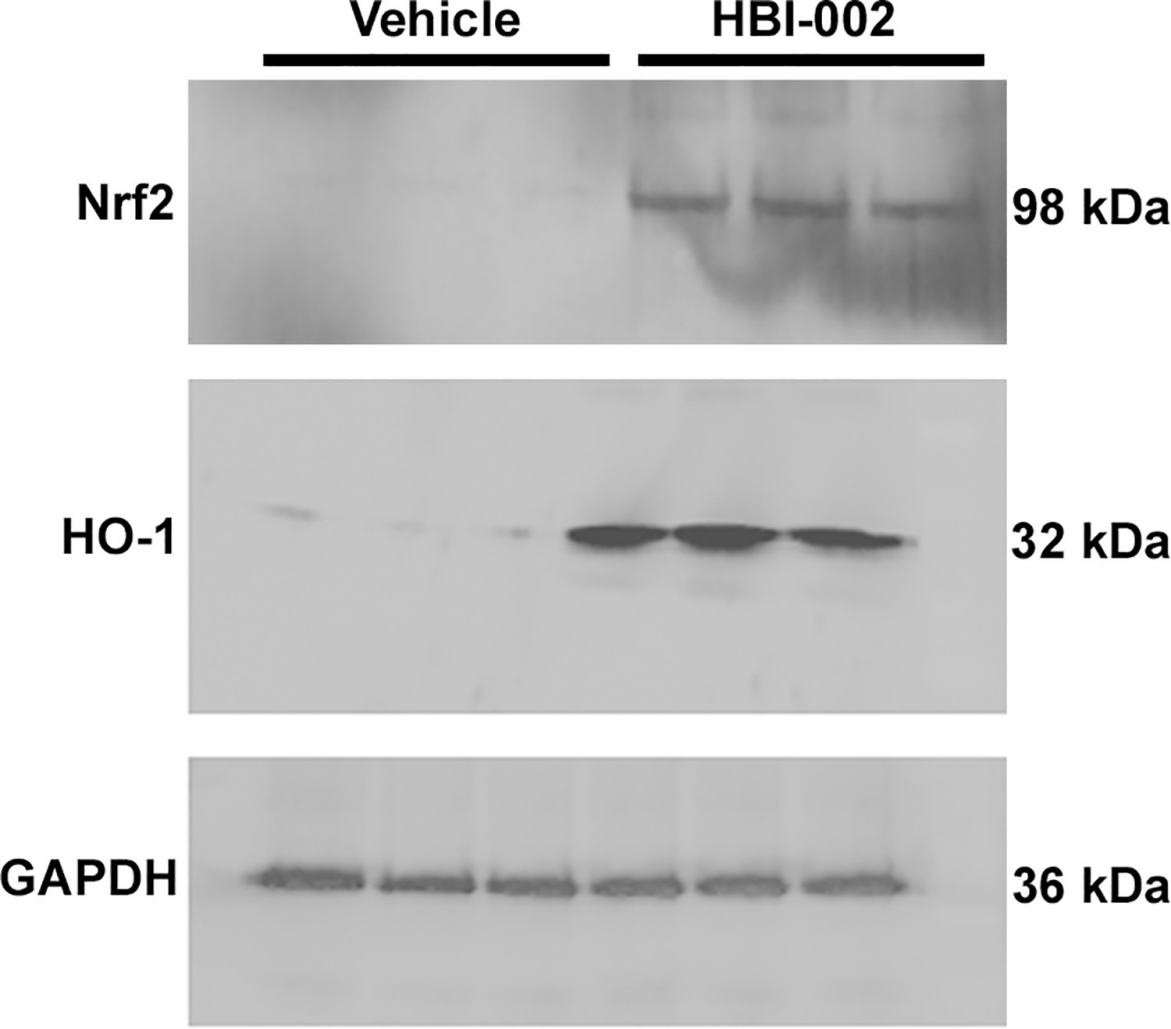
HBI-002 increases hepatic Nrf2 and HO-1 expression. Townes-SS mice (n = 3/group) were gavaged once-daily with HBI-002 or vehicle (10 ml/kg). On day 10 of treatment the livers were removed and frozen. Nrf2 and HO-1 expression was examined on immunoblots of hepatic nuclear extracts and microsomes respectively.

Livers were removed from Townes-SS mice treated once-daily for 10 days with HBI-002 or vehicle (10 ml/kg) and examined for Nrf2 and HO-1 expression by immunoblot. Hepatic Nrf2 and HO-1 were markedly increased in SS mice treated with HBI-002 compared to vehicle **(Fig 3)**. Nrf2 and HO-1 were increased 4.9-fold (p<0.01) and 12.2-fold (p<0.01) respectively (**S1 Table**), which is consistent with earlier studies showing activation of Nrf2 signaling and HO-1 induction in response to pegylated CO-Hb and CO gas [3, 29].

**Fig 4:**
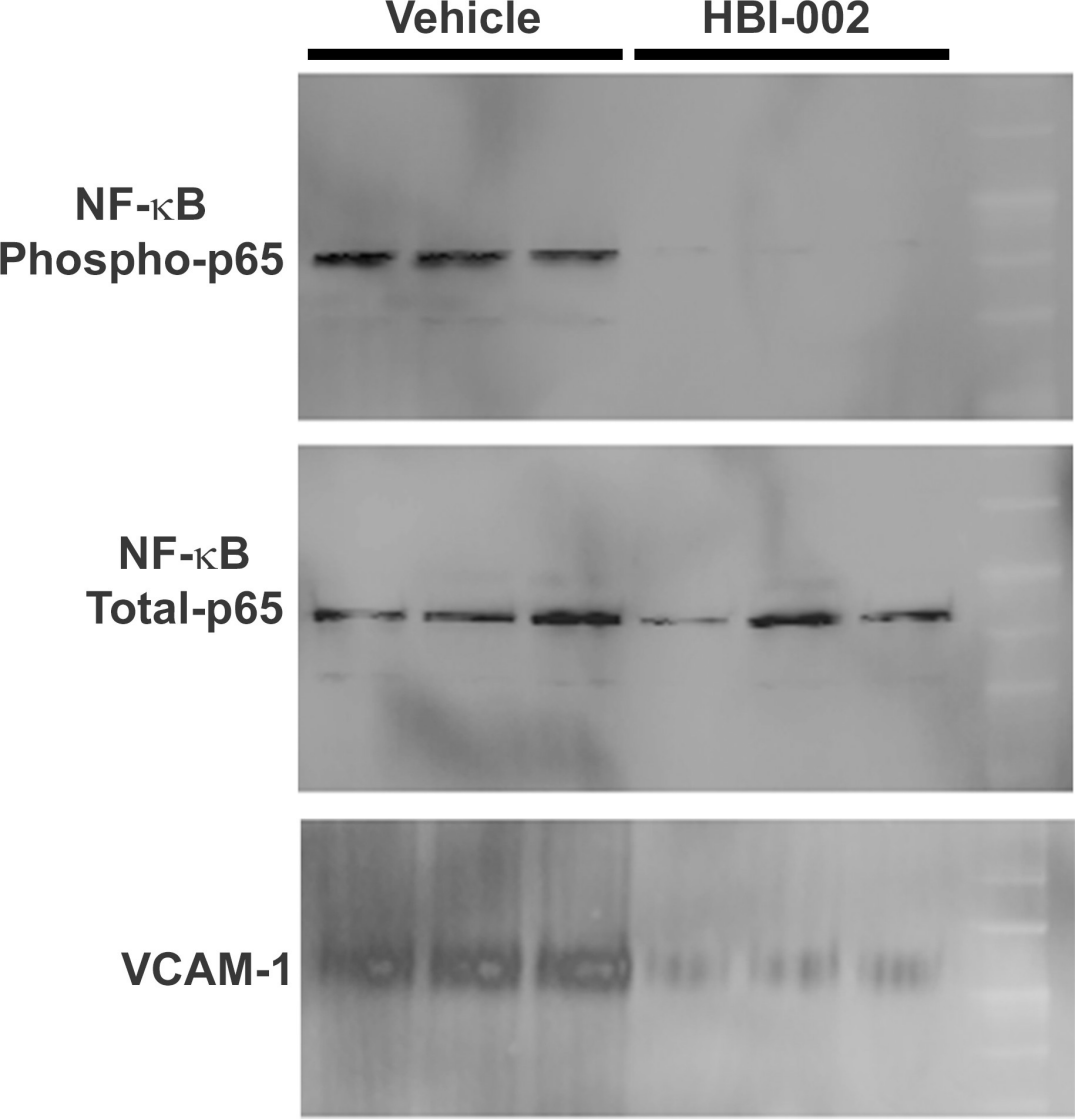
HBI-002 decreases hepatic NF-κB activation and VCAM-1 expression. Townes-SS mice (n = 3/group) were gavaged once-daily with HBI-002 or vehicle (10 ml/kg). On day 10 of treatment the livers were removed and frozen. NF-κB phospho-p65 and VCAM-1 expression was examined on immunoblots of hepatic nuclear extracts and microsomes, respectively.

Since Nrf2 and HO-1 are known to be anti-inflammatory, we also examined NF-κB activation and VCAM-1 expression in the same livers of Townes-SS mice after 10 d of gavage with HBI-002 or vehicle. Nuclear expression of NF-κB phospho-p65 is a marker of NF-kB activation [30]. Nuclear NF-κB phospho-p65 and VCAM-1 levels were decreased in the livers of SS mice treated with HBI-002 compared to vehicle-treated SS mice **(Fig 4)**. NF-κB phospho-p65 and VCAM-1 were decreased to 10% (p<0.001) and 50% (p = 0.6) respectively (**S1 Table**), of protein expression levels seen in vehicle-treated SS mice. Total NF-κB p65 levels were similar in both treatment groups. VCAM-1 is an adhesion molecule with NF-κB binding sites in its promotor. These results are consistent with CO having anti-inflammatory effects in sickle mice.

These data demonstrate that CO administered orally via HBI-002 has similar anti-vaso-occlusive and anti-inflammatory effects in SCD mice as inhaled CO and CO conjugated to PEGylated Hb [1–3]. In addition, these data suggest that HBI-002 may reduce anemia in hyperhemolytic Townes-SS mice. Additional studies with HBI-002 are needed to examine potential mechanisms relating to these anti-inflammatory effects and improvement in hemolytic parameters. We speculate that CO may have pleotropic effects on cellular mitochondria [31–33] as well as Hb S polymerization [11, 12] and RBC glutathione production [34].

## Supporting information

S1 Table Quantification of Nrf2, HO-1, NF-kB and VCAM-1 immunoblots of Townes-SS liver after 10 d treatement with Vehicle or HBI-002. Band intensities on immunoblots (Figs 3 and 4) were quantitated using ImageJ software. Values are means ± SD of vehicle and HBI-002-treated Townes-SS-mice. **P<0.01 and ***P<0.001 vehicle versus HBI-002. Treatment differences were examined using the Student’s t-test.
(PDF)

S1 Fig Nrf2 immunoblot uncropped. Townes-SS mice (n = 3/group) were gavaged once-daily with HBI-002 or vehicle (10 ml/kg). On day 10 of treatment the livers were removed and frozen. Nrf2 expression was examined on an immunoblot of hepatic nuclear extracts.
(TIF)

S2 Fig HO-1 immunoblot uncropped. Townes-SS mice (n = 3/group) were gavaged once-daily with HBI-002 or vehicle (10 ml/kg). On day 10 of treatment the livers were removed and frozen. HO-1 expression was examined on an immunoblot of hepatic microsomes.
(TIF)

S3 Fig NF-κB phospho-p65 immunoblot uncropped. Townes-SS mice (n = 3/group) were gavaged once-daily with HBI-002 or vehicle (10 ml/kg). On day 10 of treatment the livers were removed and frozen. NF-κB phospho-p65 expression was examined on an immunoblot of hepatic nuclear extracts.
(TIF)

S4 Fig NF-κB total p65 immunoblot uncropped. Townes-SS mice (n = 3/group) were gavaged once-daily with HBI-002 or vehicle (10 ml/kg). On day 10 of treatment the livers were removed and frozen. NF-κB total p65 expression was examined on an immunoblot of hepatic nuclear extracts.
(TIF)

S5 Fig VCAM-1 immunoblot uncropped. Townes-SS mice (n = 3/group) were gavaged once-daily with HBI-002 or vehicle (10 ml/kg). On day 10 of treatment the livers were removed and frozen. VCAM-1 expression was examined on an immunoblot of hepatic microsomes.
(TIF)
