# Supplementary material for: Oral carbon monoxide therapy in murine sickle cell disease: Beneficial effects on vaso-occlusion, inflammation and anemia
Source: PLoS One. 2018 Oct 11;13(10):e0205194. doi: 10.1371/journal.pone.0205194 (PMC6181332; doi:10.1371/journal.pone.0205194)
Supplement: S1 Table — Band intensities on immunoblots (Figs 3 and 4) were quantitated using ImageJ software. Values are means ± SD of vehicle and HBI-002-treated Townes-SS-mice. **P<0.01 and ***P<0.001 vehicle versus HBI-002. Treatment differences were examined using the Student’s t-test. (PDF) [file pone.0205194.s001.pdf]

S1 Table. Quantification of Nrf2, HO-1, NF-kB and VCAM-1 immunoblots of Townes-SS liver after 10 d treatement with Vehicle or HBI-002. Band intensities on immunoblots (Fig 3 and 4) were quantitated using ImageJ software. Values are means ± SD of vehicle and HBI-002-treated Townes-SS-mice. **P<0.01 and ***P<0.001 vehicle versus HBI-002. Treatment differences were examined using the Student's t-test.

(A) Nrf2	Vehicle	HBI-002
Mean Relative Expression	1.0	4.9**
SD	0.3	1.2

(B) HO-1	Vehicle	HBI-002
Mean Relative Expression	1.0	12.2**
SD	0.4	4.0

(C) NF-kB Phospho-p65	Vehicle	HBI-002
Mean Relative Expression	1.0	0.1***
SD	0.3	0.3

(D) VCAM-1	Vehicle	HBI-002
Mean Relative Expression	1.0	0.5
SD	2.7	0.4
