# Supplementary figures and images for: Oral carbon monoxide therapy in murine sickle cell disease: Beneficial effects on vaso-occlusion, inflammation and anemia
Source: PLoS One. 2018 Oct 11;13(10):e0205194. doi: 10.1371/journal.pone.0205194 (PMC6181332; doi:10.1371/journal.pone.0205194)

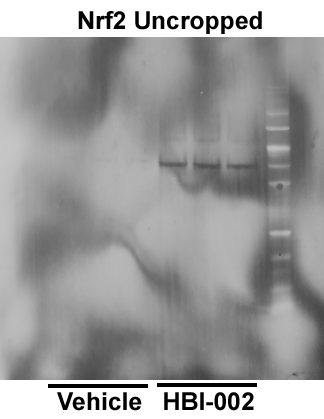

Supplement: S1 Fig — Townes-SS mice (n = 3/group) were gavaged once-daily with HBI-002 or vehicle (10 ml/kg). On day 10 of treatment the livers were removed and frozen. Nrf2 expression was examined on an immunoblot of hepatic nuclear extracts. (TIF) [file pone.0205194.s002.tif]

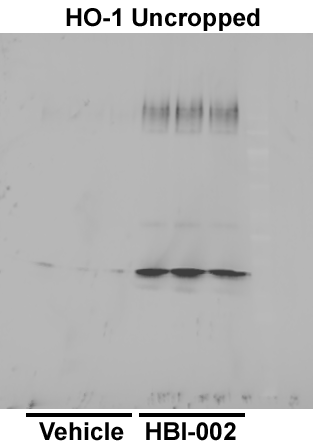

Supplement: S2 Fig — Townes-SS mice (n = 3/group) were gavaged once-daily with HBI-002 or vehicle (10 ml/kg). On day 10 of treatment the livers were removed and frozen. HO-1 expression was examined on an immunoblot of hepatic microsomes. (TIF) [file pone.0205194.s003.tif]

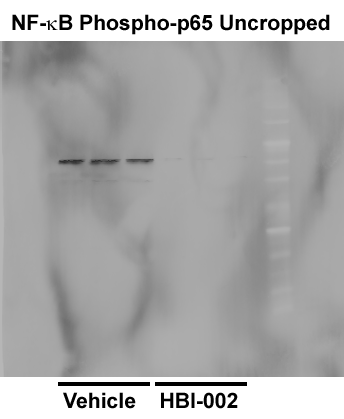

Supplement: S3 Fig — Townes-SS mice (n = 3/group) were gavaged once-daily with HBI-002 or vehicle (10 ml/kg). On day 10 of treatment the livers were removed and frozen. NF-κB phospho-p65 expression was examined on an immunoblot of hepatic nuclear extracts. (TIF) [file pone.0205194.s004.tif]

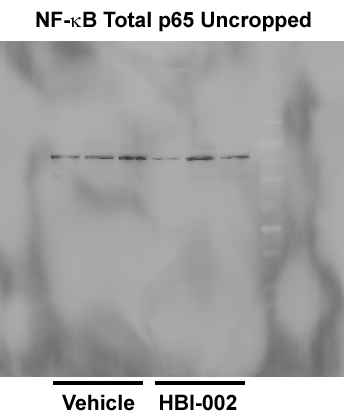

Supplement: S4 Fig — Townes-SS mice (n = 3/group) were gavaged once-daily with HBI-002 or vehicle (10 ml/kg). On day 10 of treatment the livers were removed and frozen. NF-κB total p65 expression was examined on an immunoblot of hepatic nuclear extracts. (TIF) [file pone.0205194.s005.tif]

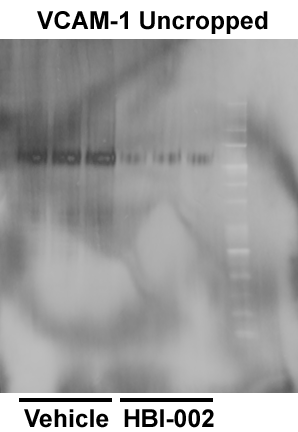

Supplement: S5 Fig — Townes-SS mice (n = 3/group) were gavaged once-daily with HBI-002 or vehicle (10 ml/kg). On day 10 of treatment the livers were removed and frozen. VCAM-1 expression was examined on an immunoblot of hepatic microsomes. (TIF) [file pone.0205194.s006.tif]
